# The effectiveness of the Healthworks Staying Steady community-based falls prevention exercise programme to improve physical function in older adults: a 6-year service evaluation

**DOI:** 10.1186/s12889-022-13832-3

**Published:** 2022-08-01

**Authors:** Emily James, Paul Oman, Michael Ali, Paul Court, Stuart Goodall, Simon J. Nichols, Alasdair F. O’Doherty

**Affiliations:** 1 Department of Sport, Exercise and Rehabilitation, Northumbria University, Newcastle-Upon-Tyne, UK; 2 Department of Mathematics, Physics and Electrical Engineering, Northumbria University, Newcastle-Upon-Tyne, UK; 3 Healthworks, Newcastle-Upon-Tyne, UK; 4 Sport and Physical Activity Research Group, Sheffield Hallam University, Sheffield, UK; 5 Advanced Wellbeing Research Centre, Sheffield Hallam University, Sheffield, UK

**Keywords:** Healthcare, Health inequality, Service evaluation, Falls, Exercise, Strength, Balance

## Abstract

**Background:**

Falls prevention exercise programmes are evidence-based and recommended for improving physical function in older adults. However, few service evaluations exist to assess the effectiveness of community-delivered interventions in practice.

**Methods:**

We conducted a six-year, retrospective evaluation of the community-delivered Staying Steady programme (Healthworks, United Kingdom). Staying Steady is a 27-week, tailored strength and balance programme delivered in a group setting (1-h, once/week) and at home (30–40 min, 2–3 times/week). Participants were referred by healthcare professionals, or self-referred, due to a history or risk of falling. Routinely collected outcome measures (30-s chair stand, Timed Up and Go, four-stage balance test, and patient reported outcomes; including ‘fear of falling’ and ‘ability to manage health’) were analysed. Factors associated with programme completion were reported. The intervention effect on physical function was analysed in subgroups: participants used arms to chair-stand or a walking-aid at both (‘aided’), neither (‘unaided’), or one assessment timepoint (‘aided at baseline only’ or ‘aided at follow-up only’).

**Results:**

There were 1,426 referrals; 835 (67.3%) participants enrolled on to the Staying Steady programme, 406 (32.7%) declined, 185 (13.0%) were inappropriately referred and excluded from analysis. After enrolling, 451 (54.0%) participants completed, and 384 (46.0%) dropped out. Chair stand performance improved in participants who were unaided (*n* = 264; median 2.0 [1.0, 4.0] repetitions; *P* < 0.001), or aided at baseline, follow-up or both (*n* = 170, *P* < 0.05). Timed Up and Go performance improved in the unaided (*n* = 387; median ˗3.1 [˗5.4, ˗1.4] s, *P* < 0.001), and aided at baseline only (*n* = 32; median ˗4.9 [˗10.8, ˗3.4] s, *P* < 0.001) groups. Four-stage balance performance improved (*n* = 295; median 1.0 [0.0, 1.0] points, *P* < 0.001). After programme completion, participants self-reported an improved ability to manage their health and daily activities, improved confidence, and a reduced fear of falling. Presence of chronic obstructive pulmonary disease, fear of falling, prescribed nutritional support, disability and social deprivation influenced non-completion of Staying Steady.

**Conclusions:**

Completing Staying Steady improved physical function in older adults. Methods to encourage retention of participants from groups associated with low uptake and adherence should be investigated.

**Supplementary Information:**

The online version contains supplementary material available at 10.1186/s12889-022-13832-3.

## Introduction

Deteriorating muscle strength and physical function increases the incidence of falling, hospitalisation [1] and healthcare costs in older adults [2]. In the United Kingdom (UK) and the United States of America (USA), guidelines recommend the implementation of multifactorial interventions to prevent falls in older people, including an individually prescribed strength and balance exercise program [3–5]. When delivered using best practice protocols, Public Health England estimates that evidence-based falls prevention programmes generate a societal return on investment (i.e. health and social care-related monetary savings plus gains in quality adjusted life years, compared to usual care) of between £1.97 and £7.43, per £1.00 of delivery costs [6]. Furthermore, pooled evidence from randomised controlled trials supports the use of exercise interventions to improve muscle strength, balance, and gait speed [7] and reduce incidence of fall in adults aged ≥ 60 years [8]. These data are promising; however, few service evaluations have assessed the effectiveness of these interventions in the UK [9–12] or globally [13]. Importantly, evaluation of community-delivered exercise programmes at a local level, using routinely collected data, has greater ecological validity than data collected in randomised controlled trials [14].

Staying Steady is a community-delivered falls prevention programme in the North-east of England, adapted from the Falls Management Exercise (FaME) intervention [15, 16]. Staying Steady initially included four eight-week blocks, alternating group-based and home-based exercise sessions [17]. A small (*n* = 5) mixed methods evaluation of this delivery format reported Staying Steady group session adherence of ~ 80% and positive narrative accounts from the participants, citing improved mental and physical health [17]. Participant reports coincided with objectively measured improvements in strength, balance and physical function, however, statistical analyses were not performed [17]. Currently, Staying Steady consists of one-hour group-based sessions delivered once per week over 27 weeks. To help participants meet the 50 h of exercise recommended to reduce fall risk [18], group-based sessions are supplemented with home exercises to be completed two to three times per week, for a maximum of 30 to 40 min per session. The effectiveness of the current Staying Steady programme in improving outcomes related to physical function, goal setting, and factors associated with attrition, requires evaluation in a larger cohort. This would enable identification of strengths and weaknesses of the programme and may provide an evidence base for more widespread implementation of community-run falls prevention exercise programmes. The aim of this single centre retrospective service evaluation, conducted in the North-east of England, was to assess the effectiveness of the Staying Steady programme in practice, to improve physical function and assess patient reported outcome measures (PROMs) and factors associated with attrition in older adults who are at risk of falling.

### Objectives

#### Primary objective

- Evaluate the effectiveness of the Staying Steady exercise programme at a local level to improve physical function (30-s chair stand [CS], timed up and go [TUG], and four-stage balance test [4SBT] performance) in older adults at risk of falling.

#### Secondary objectives

- Evaluate adherence to, attrition from, and safety of, the Staying Steady exercise programme.
- Understand whether baseline demographics impacted attrition and outcome variables.
- Evaluate PROMs, goal setting and participant evaluation of the programme.

## Methods

### Study design

This is a non-experimental, retrospective service evaluation. Healthworks is a community health charity, independent to the National Health Service (NHS), commissioned by multiple organisations including Newcastle City Council and the Newcastle upon Tyne Hospitals (NUTH) NHS Foundation Trust. The Staying Steady programme was first commissioned in April 2010 through the NUTH Trust. Data were collected between January 2015 and April 2021 by Healthworks practitioners as part of standard service delivery of Staying Steady and for audit and evaluation. Participant data were anonymised, stored and analysed by the research team on Healthworks property. Ethical approval was provided by the Northumbria University Health and Life Sciences ethics committee (reference 34,401). Permission was granted by Healthworks to audit, evaluate and publish these data. Participants gave informed consent for Healthworks to store their data for monitoring and evaluation purposes. The academic team ensured that legal and ethical standards were met by performing the evaluation in collaboration with Healthworks and in accordance with guidance from the general data protection regulation (GDPR) (Article 89.1) and National Institute for Health Science Research UK (NIHR) [19]; namely, the processing of healthcare data without consent is permitted for scientific or statistical reasons if data are anonymised and unidentifiable.

### Sample

Anonymised secondary data from participants at five community leisure centres in four locations in the North-east of England were included for analyses. Participants with a documented referral to the Healthworks “Staying Steady” community programme between January 2015 and April 2021, were included in this service evaluation. Data were extracted from records at Healthworks between May 2021 and September 2021.

Participants registered at a Newcastle upon Tyne GP could be referred based on one or more of the following criteria:

- Feel unstable and unbalanced
- Fear of falling
- History of falls
- Low bone density and / or family history of osteoporotic fracture

**Fig. 1:**
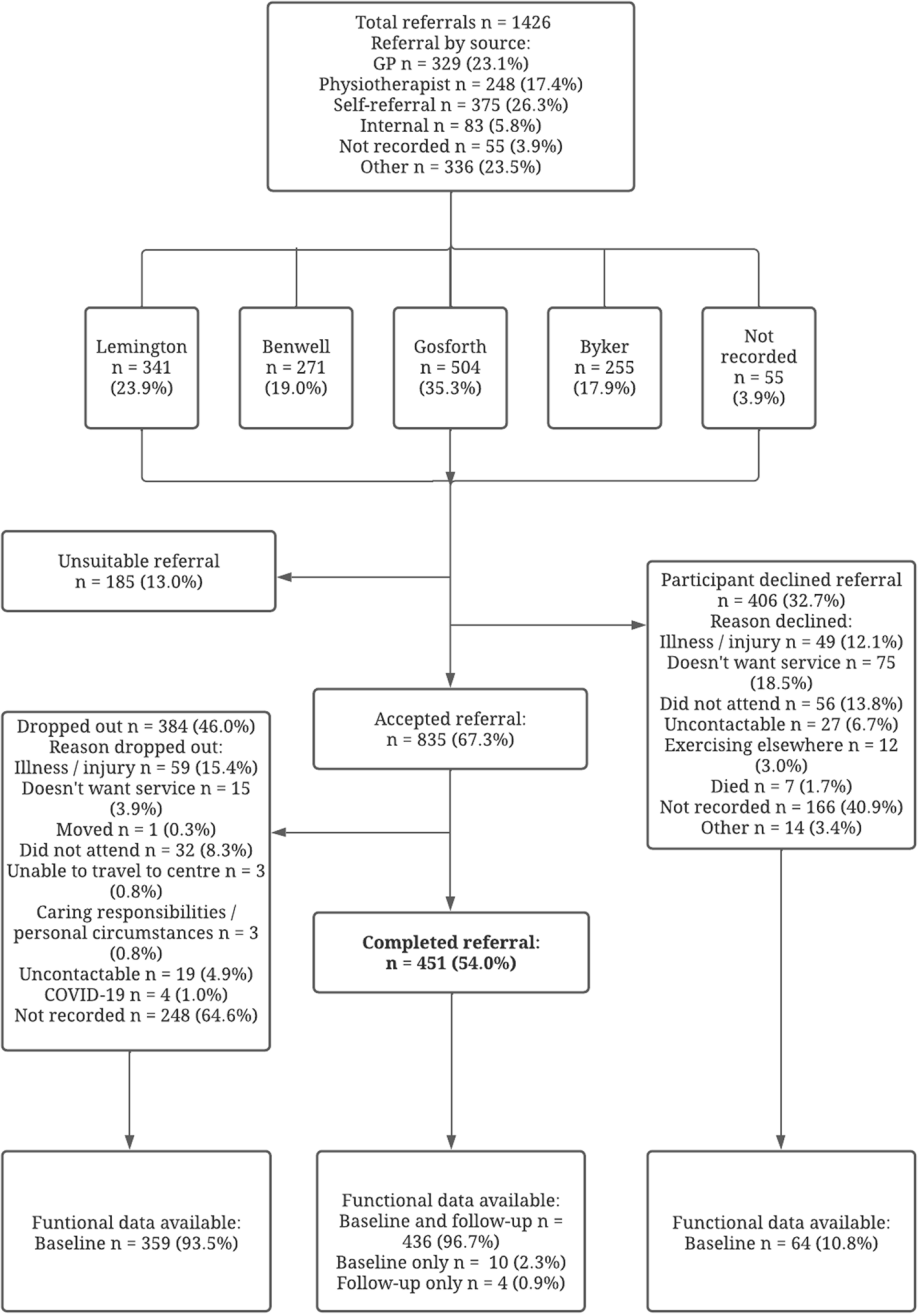
Flowchart of referrals to the Staying Steady falls prevention exercise programme at Healthworks Newcastle, between January 2015 and April 2021. Frequencies are represented as a percentage of the number of participants in the level above, except where individual reasons for declines and dropouts are listed. These represent percentages of the participants who declined or dropped out, respectively

Participants needed to be able to mobilise independently with or without the use of a walking aid and have the cognitive ability to follow instructions. Staying Steady practitioners used the Falls Risk Assessment Tool (FRAT; [20]), functional ability and medical history records to confirm the participants suitability. Safety to participate was continually assessed by trained practitioners during their first three exercise sessions. Participants withdrawn from the programme due to safety concerns during early exercise sessions or assessments were considered an ‘unsuitable referral’ (Fig. 1). For example, participants might be withdrawn and referred elsewhere due to a medical condition that contraindicates exercise [21] or very poor, deteriorating physical function. Other examples of unsuitable referrals include where the Staying Steady referral criteria have not been met, or where the referred person would benefit from a more advanced exercise programme due to having a higher fitness level than the target Staying Steady participant.

### Intervention

Staying Steady is an individually tailored 27-week group exercise programme involving one-hour sessions, weekly (Table 1). Group exercise sessions were led by exercise practitioners at a community health charity (Healthworks, UK). Delivery of the programme was in fixed blocks, i.e., Staying Steady started Week One on a set date and continued for the next 27-weeks. After the 27-week programme was delivered, Staying Steady started again at Week One for new referrals. It was not essential that new participants enrolled at Week One, they could join at any time. However, these participants still finished on Week 27 and therefore had a shorter programme duration. Exercise sessions started with a 10-min warm up, followed by aerobic, strength and balance exercises. Alternative lower intensity options, typically chair-based exercises, were provided for particularly deconditioned participants, the need for this was subjectively determined by a trained exercise practitioner. Initially, the different exercise modes were completed separately, allocating approximately 10 min each to aerobic, strength and balance training. Later, aerobic, strength and balance exercises were combined in a circuit. Progression was achieved by increasing the number of repetitions, the amount of time completing an exercise or the number of rounds in a circuit. More difficult exercises, such as press-ups, tandem or single-leg stands were introduced as participants progressed through the programme. All exercise sessions ended with a cool down and stretching. To support participants to meet the recommended 50-h dose of exercise [18], similar progressive home-based exercises were prescribed. Home-based exercises were recommended to be performed two to three times per week, for 10 to 20 min per session, and progress to a maximum of 30 to 40 min per session. The Staying Steady programme included two practitioner delivered education sessions. The first education session (week nine) covered fall risk factors, risk reduction and recovery strategies. Content from the first education session was reiterated in a second education session (week 18), and participants were given information about local exercise programmes to encourage long-term exercise engagement after completion of Staying Steady.

**Table 1:** Overview of the Healthworks Staying Steady 27-week falls prevention exercise programme

Time point	Duration	Exercise / activity
Baseline assessments		• 30-s chair stand • Timed up and go • Four-stage balance test • Patient reported outcome measures • Goal setting
All exercise sessions	10 min 10 min	• Warm-up with mobility exercises • Cool down
Exercise 1 to 8	5 to 10 min each	• Aerobic • Strength • Balance
Education 1	60 min	• Fall risk factors and fall recovery
Exercise 9 to 16	8 to 10 min each	• Aerobic • Strength • Balance
Education 2	60 min	• Summary of Education 1 • Continuing exercise after Staying Steady
Exercise 17 to 24	15 to 20 min	• Aerobic and strength circuits (6 exercises, 2–3 rounds)
	6 min	• Balance exercises
Follow-up assessments		• 30-s chair stand • Timed up and go • Four-stage balance test • Patient reported outcome measures • Participant self-evaluation of progress and evaluation of the Staying Steady programme

Baseline and follow-up outcome measures collected as part of standard practice and typical group-based exercise session content are detailed

### Outcomes

Outcome measures were assessed at baseline and 27-weeks (Table 1). Goal setting and evaluation questionnaires were developed in-house and PROMs were adapted from the Patient Reported Outcome Measures in England Data Dictionary version 3.4 [22]. Outcome data are missing for some participants due to the retrospective study design and changes to Healthworks’ routine data collection around 2017. For evaluation purposes, where previously assessed items were later removed from standard practice, these variables were excluded from analysis or grouped with the most similar equivalent in the updated format (Supplementary Material 1, eTable 1 and eTable 2 in Additional File 1).

#### Participant characteristics

Participants who self-referred reported presence of disability, medication, balance and functional ability, falls history and history of collapse, adapted from the FRAT [20]. Where available, a full medical summary, provided in the referral, was used to report participant characteristics. When this was unavailable, a patient-reported medical history related to cardiovascular, pulmonary, musculoskeletal, neurological and psychological issues from an in-house triage questionnaire was used. Participant age (years), sex, postcode (socioeconomic index), stature (m), mass (kg), medication and medical history were recorded. The term cardiac disease refers to any heart-related medical condition reported in the medical summary or medical history. Risk for coronary heart disease (CHD) was defined by presence of ≥ 1 risk factor, including diabetes, hypertension, or dyslipidaemia, in the absence of a cardiac diagnosis.

#### 30-s chair stand (CS) test

Participants completed as many CSs as possible in 30 s, without using their arms for support (unaided) [23]. If necessary, participants pushed themselves into a standing position using their hands on the chair or a walking aid (aided). The 30-s CS test is a measure of physical function and proxy for leg strength assessment in older adults [23]. Community-dwelling older adults who complete < 11 repetitions in 30 s are at increased risk of falling [24].

#### Timed up and go test (TUG)

The time taken to rise from a chair, walk three metres in a straight line, turn, and return to the seated start position is recorded in seconds [25]. Where possible, participants performed the TUG without using their arms or a walking aid (unaided). If necessary, participants used a walking aid, pushed themselves into a standing position or used the wall for balance (aided). Inability to complete the TUG in < 15 s is associated with increased risk for hospitalisation, difficulty in activities of daily living and multiple falls, compared to older adults who completed the TUG in ≤ 12 s [26].

#### Four-stage balance test (4SBT)

The 4SBT comprises four foot positions, held up to 10 s each: (1) parallel, (2) semi-tandem, (3) tandem, (4) and one-legged stance [27]. The highest level held for 10 s was recorded as the participants score. The 4SBT was included as an outcome measure by Healthworks from 2017 onwards, explaining the lower number of cases for this variable relative to the other primary outcome measures. Inability to complete the tandem stand (stance 3) for 10 s indicates increased fall risk falling [28].

#### Patient reported outcome measures

Participants selected a response from a five-point Likert-scale to the following prompts: (1) how I feel about managing my health, (2) How I feel about managing my daily activities, (3) my fear of falling, (4) my confidence when walking outside, and (5) my social network, adapted from the Patient Reported Outcome Measures in England Data Dictionary version 3.4 [22]. Some participants selected more than one response from the Likert scale; here the lowest number response was recorded for analysis. See eTable 1 (Additional File 1) for previous iterations of the PROMs questionnaire used by Healthworks.

#### Goal setting and evaluation

Goal setting questionnaires were developed in-house by Healthworks (Supplementary Material 1 in Additional File 1). Before 2017 participants could select one primary goal from the list. From 2017, participants chose as many goals as they wished from an amended list. Questionnaires provided to participants were updated during changes to standard delivery of the programme, implemented around 2017 with the approval of a steering group and commissioners of the community health charity (Healthworks).

Participants reviewed the programme and their self-reported progress in a final in-house questionnaire (eTable 2 in Additional File 1).

### Data analysis

Anonymised data from Healthworks records were transferred to a spreadsheet (Microsoft Excel, Office 365) by EJ. Statistical analyses were performed using SPSS (v27, IBM, Chicago, USA). Histograms and QQ-plots were visually assessed to determine the distribution of data. Categorical data are reported as frequency and percentage. Quantitative descriptive statistics are reported as median and interquartile range (IQR). Wilcoxon signed-rank tests assessed pre- to post-intervention change in CS, TUG, 4SBT performance, PROMs, and sub-analyses of primary outcomes (1) following removal of participants with the least (1^st^ decile) and most (10^th^ decile) amount of time in weeks between baseline and follow-up assessments, and (2) by sub-groups of chronic diseases. All outcomes were assessed using complete case analysis (pairwise deletion) because: imputation of outcome data can distort the results [29], missing TUG and CS results for completers were negligible (< 5%) [30], and the absence of variables was independent of their value (“missing completely at random”) and will not introduce bias to the analyses [30, 31]. The intervention effect on physical function was analysed and reported by grouping participants; ‘aided’ if they used arms to chair-stand or used a walking-aid, or ‘unaided’ if not. Participants who used arms to chair-stand or used a walking-aid at baseline but not follow-up are described as ‘aided at baseline only’, and where the reverse was true ‘aided at follow-up only’. The minimal detectable change (MDC) value for the 30-s CS and the TUG are 3.9 repetitions [32] and 1.8 s [33] in older adults, respectively. Statistical significance was set at *P* < 0.05.

Index of Multiple Deprivation (IMD) was calculated from address postcodes using 2019 UK Government data [34]. The IMD rank is reported in national reference deciles, where deciles one and 10 represent the most and least deprived 10% of areas, respectively [35]. To create a categorical value, IMD deciles were dichotomised at the median to create two groups of high (< 5^th^ decile) or low (≥ 5^th^ decile) deprivation. Using postcodes, the distance (km) between the participants addresses and the Healthworks centre to which they were referred was calculated using an online tool (freemaptools.com).

We identified three potential outcomes following referral to Staying Steady: declined (no attendance), dropout (attended ≥ 1 Staying Steady session and dropped out before registering any follow-up outcome measures), and complete (present until the end of the programme and completed ≥ 1 follow-up outcome measure). We investigated the influence of baseline characteristics on starting and/ or completing Staying Steady, using Chi-squared (Χ^2^; categorical variables), Mann Whitney-U tests (continuous and ordinal variables) and binomial regression. Declined potential participants who were inappropriately referred (detailed in Sect. 2.2) were removed from the analysis as they do not represent the target cohort for this evaluation. The effect size for significant associations is reported using Phi (ϕ), interpreted as follows: very strong (ϕ > 0.25), strong (ϕ > 0.15 and ≤ 0.25), moderate (ϕ > 0.10 and ≤ 0.15), weak (ϕ > 0.05 and ≤ 0.10), or no association (ϕ ≤ 0.05; [36]). Stepwise binomial logistic regression with backwards elimination based on the likelihood ratio was performed to assess factors influencing the referral outcome when grouped as completers versus non-completers (dropouts and declined referrals, both separately and combined). Likelihood ratio is the strongest test for the statistical contribution of individual variables to a model and is preferred over the Wald statistic where continuous independent variables are investigated [37]. All baseline variables were included in the full regression models, excluding those with a substantial amount of missing data (> 40%; body mass index [BMI], self-referral questions [detailed in Table 2], goal setting) [30]. The Box-Tidwell Test confirmed the assumption of linearity between the continuous independent variables (age, IMD rank and distance from venue) and the logit of the outcome. Other assumptions of logistic regression (binary dependent variable and independence of observations) were met. Relative risk (RR) with 95% confidence interval (CI) was calculated for interactions from the logistic regression, using 2 × 2 contingency tables of outcome against covariates.

**Table 2:** Baseline demographics of participants referred to the Staying Steady 27-week falls prevention programme

Variable	All		Completers		Non-Completers		*P*-value ^a^	*P*-value ^b^
	n	% or median (IQR)	n	% or median (IQR)	n	% or median (IQR)		
Age (years)	1417	80.0 (73.0, 85.0)	448	80.0 (74.0, 84.0)	969	80.0 (72.0, 86.0)	0.879	0.810
Female	1000	70.1%	327	72.5%	673	69.0%	0.182	0.283
Body mass index (kg/m^2^)	526	26.8 (23.8, 31.2)	179	27.2 (23.8, 32.1)	347	26.6 (23.7, 30.9)	0.653	0.481
Index of Multiple Deprivation (decile)	1370	5.0 (2.0, 8.0)	437	6.0 (3.0, 9.0)	933	4.0 (2.0, 8.0)	< 0.001**	< 0.001**
Distance from programme venue (km)	1308	1.87 (1.14, 2.68)	436	1.81 (1.12, 2.81)	873	1.89 (1.17, 2.65)	0.731	0.435
Ethnicity	851							
White (any White background)	809	95.1%	264	96.0%	545	94.6%	0.820	0.767
Mixed British	12	1.4%	3	1.1%	9	1.6%		
Other mixed background	6	0.7%	2	0.7%	4	0.7%		
Asian / Asian British	24	2.8%	6	2.2%	18	3.1%		
**Disability**								
Registered as disabled	304	29.0%	112	25.6%	192	31.5%	0.038*	0.140
Self-identify as disabled	477	46.0%	198	45.1%	279	46.7%	0.603	0.840
Mobility-related disability	411	39.2%	165	37.6%	246	40.3%	0.369	0.555
Hearing-related disability	142	13.5%	63	14.4%	79	12.9%	0.507	0.504
Sight-related disability	86	8.2%	36	8.2%	50	8.2%	0.990	0.818
Learning-related disability	15	1.4%	6	1.4%	9	1.5%	0.884	0.788
Other disability	49	4.7%	22	5.0%	27	4.4%	0.652	0.596
**Medical history**	1264							
Cardiac disease	430	34.5%	128	31.8%	302	35.8%	0.171	0.256
Heart failure	55	4.4%	15	3.3%	40	4.7%	0.418	0.307
Coronary heart disease	243	17.0%	78	19.4%	165	19.5%	0.951	0.948
At risk for coronary heart disease	532	42.7%	175	43.5%	357	42.3%	0.681	0.912
Stroke / transient ischemic attack	251	20.1%	68	16.9%	183	21.7%	0.050	0.070
Chronic obstructive pulmonary disease	108	8.7%	18	4.5%	90	10.7%	< 0.001**	< 0.001**
Osteopenia / osteoporosis	240	16.8%	75	18.7%	165	19.5%	0.709	0.847
Chronic kidney disease	374	26.2%	119	29.6%	255	30.2%	0.826	0.949
**Medication**	1290							
Cardiac glycoside	32	2.5%	13	3.9%	19	2.2%	0.324	0.223
ACE inhibitor	329	25.5%	99	23.6%	230	26.5%	0.264	0.241
Statin	761	59.0%	254	60.5%	507	58.3%	0.452	0.578
Beta-blocker	348	27.0%	104	24.8%	244	28.0%	0.213	0.224
Nitrates	139	10.8%	39	9.3%	100	11.5%	0.231	0.253
Oral nutrition support	23	1.8%	1	0.2%	22	2.5%	0.004**	0.003**
**Self-referral questions**								
Falls in the last 12 months (n)	514	1 (0,3)	188	1 (0,3)	326	1 (0,3)	0.842	0.773
Prescribed ≥ 4 tablets per day	539							
Yes	416	77.2%	144	72.7%	272	79.8%	0.061	0.156
Presence of balance issues	536							
Yes	502	93.7%	185	93.9%	317	93.5%	0.855	0.578
Able to rise from a chair unaided	532							
Yes	253	47.6%	98	50.0%	155	46.1%	0.389	0.691
History of blackout in previous 12 months	526							
Yes	66	12.5%	27	13.8%	39	11.8%	0.490	0.103
Able to stand unaided for five minutes	44							
Yes	30	68.2%	3	60.0%	27	69.2%	0.677	0.845
**Goal setting**	782							
Reduce fear of falling	456	58.3%	233	57.2%	223	59.5%	0.530	0.893
Feel stronger	453	57.9%	220	54.1%	233	62.1%	0.022*	0.031*
Feel more stable	578	74.0%	299	73.5%	279	74.6%	0.718	0.862
Feel more confident out and about	531	68.0%	270	66.3%	261	69.8%	0.302	0.682
Socialise more	219	28.0%	100	24.6%	119	31.7%	0.026*	0.078
Feel fitter	52^c^	27.1%	37	29.8%	15	22.1%	0.246	0.270
Feel more able to manage my health	21^c^	10.9%	17	13.7%	4	5.9%	0.097	0.103

Number of cases are listed for individual variables due to missing data. "Non-completers" represents declined referrals and those who dropped out following ≥ 1 session. “Completers” were present until the end of the programme and registered ≥ 1 follow-up outcome measure. *IQR* Interquartile range. ^a^ *P*-value for difference between completers and non-completers, including all referrals. ^b^ *P*-value for difference between completers and non-completers, after removal of non-completers who were inappropriately referred (*n* = 185). ^c^ *n* = 192 for this outcome. *P < 0.05 **P < 0.01

#### Sample size

This was a retrospective service evaluation and therefore the sample was determined by the number of documented referrals and participant records in the evaluation period. The sample is an outcome of the service evaluation.

## Results

### Participant demographics

During the evaluation period, 1,426 referrals were made to Staying Steady, of which 13.0% (*n* = 185) were considered an unsuitable referral. Of the remaining 1241 referrals, 32.7% (*n* = 406) were declined by the participant (Fig. 1). Of the 835 participants who joined Staying Steady, 54.0% (*n* = 451) completed the 27-week programme. Baseline demographics of the referred participants are shown in Table 2. Due to missing data, the number of participants is listed for individual variables.

### Primary outcomes

#### 30-s chair stand (CS) test

**Fig. 2:**
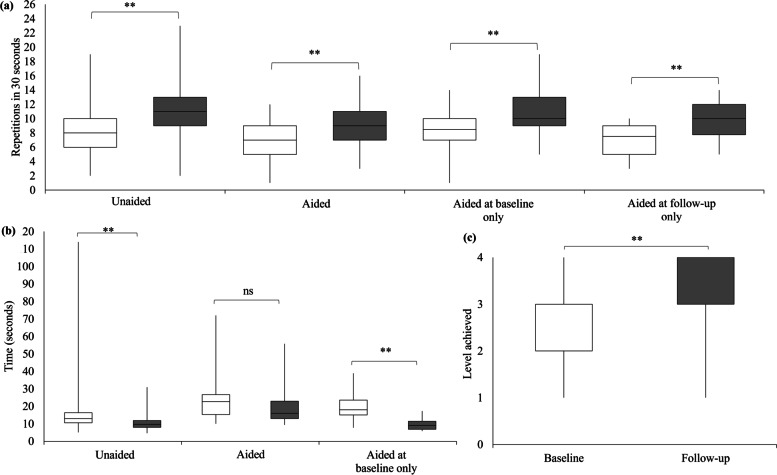
Baseline (white box plots) and follow-up (grey box plots) primary outcome data for the (**a**) 30-s chair stand, (**b**) Timed up and go, and (**c**) Four-stage balance test. Participants were grouped based on whether they used arms to chair-stand or used a walking-aid at both timepoints (‘aided’), neither timepoint (‘unaided’), at baseline but not follow-up (‘aided at baseline only’), or at follow-up but not baseline (‘aided at follow-up only’). Box plots represent the median, 25^th^ and 75.^th^ percentile. Vertical lines represent minimum and maximum values. **P < 0.001; ns = not significant at *P* < 0.05. Numerical values shown in Table 3

There was an increase in the number of repetitions completed by participants who performed the test unaided (*n* = 264, 60.8%), aided (*n* = 54, 12.4%), aided at baseline only (*n* = 94, 21.7%) and aided at follow-up only (*n* = 22, 5.1%; Fig. 2A). An improvement in the number of CS repetitions greater than the MDC (≥ 3.9 repetitions; [32]) was achieved by 36.0% (*n* = 95), 24.1% (*n* = 13), 28.7% (*n* = 27) and 40.9% (*n* = 9) of participants who completed the 30-s chair stand unaided, aided, aided at baseline only, and aided at follow-up only, respectively.

#### Timed up and go test (TUG)

Time to complete the TUG improved in participants who were unaided (*n* = 387, 89.2%) and aided at baseline only (*n* = 32, 7.4%), but not in participants who were aided at both timepoints (*n* = 13, 3.0%; Fig. 2B). Two participants (0.5%) completed the TUG unaided at baseline and aided at follow-up and were not analysed. A reduction in TUG time greater than the MDC (≥ 1.8 s; [33]) was achieved by 70.0% (*n* = 271), 53.8% (*n* = 7) and 93.8% (*n* = 30) of participants performing the test unaided, aided, and aided at baseline only, respectively.

#### Four-stage balance test (4SBT)

There was a median improvement in the score achieved in the 4SBT for 295 participants (Fig. 2C).

### Secondary outcomes

#### Sub-analyses of primary outcomes based on time between assessments

The median time between baseline and follow-up assessments for primary outcomes was 25.0 weeks (IQR 24.0, 26.0 weeks; minimum 9.0 weeks; maximum 40.0 weeks). Participant referrals did not always align with the beginning of a 27-week programme. Therefore, participants might have joined an ongoing programme mid-way through or completed their baseline assessments before waiting for a new programme to start, explaining the variation in time between the two assessments. Some functional data was recorded prior to the participants referral date by the referring agency or person, such as a physiotherapist. Repeating the analysis after removal of participants from the first (≤ 20.7 weeks), tenth (≥ 29 weeks) or unknown decile for time between assessments (*n* = 119) did not change the significance of the findings (Table 3).

**Table 3:** Sub-analyses of primary outcomes measures by time between assessments for completers of the Staying Steady programme

		**Excluding participants from the first (≤ 20.7 weeks) and tenth decile (≥ 29 weeks) for time between assessments **^**a**^			**All completers**			
**Outcome measure**	**n**	**Baseline**	**Follow-up**	***P*-value**	**n**	**Baseline**	**Follow-up**	***P*-value**
Chair stand (repetitions)								
*Unaided*	196	8.0 (6.0, 10.0)	11.0 (9.0, 13.0)	< 0.001**	264	8.0 (6.0, 10.0)	11.0 (9.0, 13.0)	< 0.001**
*Aided*	37	7.0 (5.5, 9.0)	9.0 (8.0, 11.0)	< 0.001**	54	7.0 (5.0, 9.0)	9.0 (7.0, 11.0)	< 0.001**
*Aided at baseline only*	64	8.5 (6.3, 11.0)	11.0 (9.0, 13.0)	< 0.001**	94	8.5 (7.0, 10.0)	10.0 (9.0, 13.0)	< 0.001**
*Aided at follow-up only*	18	6.0 (5.0, 8.3)	10.0 (7.8, 11.3)	< 0.001**	22	7.5 (5.0, 9.0)	10.0 (7.8, 12.0)	< 0.001**
Timed Up and Go (s)								
*Unaided*	280	13.0 (10.5, 16.0)	9.7 (8.0, 11.5)	< 0.001**	387	13.0 (10.5, 16.4)	9.7 (8.0, 11.9)	< 0.001**
*Aided*	10	21.0 (15.4, 33.3)	16.5 (15.5, 20.9)	0.344	13	22.7 (15.3, 26.7)	16.0 (13.0, 23.0)	0.221
*Aided at baseline only*	23	18.0 (14.8, 21.1)	12.3 (10.0, 14.3)	< 0.001**	32	18.0 (15.0, 23.6)	12.3 (10.0, 14.6)	< 0.001**
Four-stage balance test (level)	225	2.0 (2.0, 3.0)	3.0 (3.0, 4.0)	< 0.001**	295	2.0 (2.0, 3.0)	3.0 (3.0, 4.0)	< 0.001**

Values are median (interquartile range). Participants were grouped based on whether they used arms to chair-stand or used a walking-aid at both timepoints (‘aided’), neither timepoint (‘unaided’), at baseline but not follow-up (‘aided at baseline only’), or at follow-up but not baseline (‘aided at follow-up only’). ^a^ The amount of time between baseline and follow-up assessments was calculated for all completers of Staying Steady. Sub-analyses of primary outcome measures were performed following removal of participants from the first and tenth decile, for the amount of time between assessments. Primary outcome data from all completers are shown in the right-hand column for direct comparison. **P < 0.01 between baseline and follow-up

#### Sub-analyses of primary outcomes based on chronic diseases

Medical history was available for 390 (89.4%) completers with pre- and post-intervention data for at least one primary outcome. Most improvements in primary outcomes remained when stratified by chronic disease presence (eTable 3 in Additional File 1). Fewest improvements are reported in participants with heart failure (HF), and with chronic obstructive pulmonary disease (COPD).

#### Adherence, attrition, and adverse events

Attendance at each session, as a percentage of the number of expected participants, was 76, 80 and 83% during the period 2019–20, 2018–19 and 2017–8, respectively. Attrition was 46.0% (Fig. 1.) No illness or injury related to the intervention were recorded during the data collection period.

#### Factors associated with referral outcome

##### Completers versus non-completers

Baseline demographics for completers and non-completers are shown in Table 2. There was no difference in age, proportion of female participants, BMI, distance from the Staying Steady programme venue, ethnicity, or nature of disability (where present), between completers and non-completers. After removal of inappropriate referrals (*n* = 185), non-completion was associated with a higher incidence of prescribed oral nutritional support (ϕ = ˗0.089, *P* = 0.003), COPD diagnosis (ϕ = ˗0.110, *P* < 0.001), higher deprivation (ϕ = ˗0.103, *P* < 0.001) and setting a goal of feeling stronger (ϕ = ˗0.079, *P* = 0.031).

Binomial logistic regression of completion versus non-completion, where non-completion combined dropouts and declined referrals, captured 31.3% (*n* = 389) of selected cases. Following backwards elimination four variables remained in the final model: prescribed oral nutritional support (*P* = 0.999), fear of falling (*P* = 0.005), history of stroke or transient ischaemic attack (*P* = 0.062) and COPD (*P* = 0.012). The model was significant (*P* < 0.001), explained between 8 (Cox & Snell R square) and 11% (Nagelkerke R squared) of variation in completion status, and accurately classified 64% of cases. Non-completers were more likely than completers to be prescribed oral nutritional support (*n* = 1135; RR 11.16, 95% CI 1.50, 83.07), and diagnosed with COPD (*n* = 1092; RR 2.43, 95% CI 1.47, 4.00). When baseline fear of falling was classed as high (Likert scale responses one or two) or low (Likert scale responses four or five; detailed in Table 4), non-completers were more likely than completers to have a high fear of falling (*n* = 608; RR 1.52, 95% CI 1.19, 1.94). The RR for history of stroke or transient ischaemic attack was not significant (*n* = 1092; RR 1.27, 95% CI 0.98, 1.64).

**Table 4:** Patient reported outcomes from the Healthworks Staying Steady 27-week falls prevention programme

Domain	Baseline (All)	Baseline (Completers only)	Follow-up (Completers only)	Change
	Median response (IQR) N (%) for individual responses			*P*-value
**How I feel about managing my health**				
Total responses	827	419	406	
Median response	4.0 (3.0, 5.0)	4.0 (3.0, 5.0)	4.0 (3.0, 5.0)	< 0.001**
1. “I don’t feel able to manage”	13 (1.6)	4 (1.0)	6 (1.5)	
2. “It’s a struggle and I get a lot of help”	68 (8.2)	30 (7.2)	11 (2.7)	
3. “I get some help from other people”	231 (27.9)	109 (26.0)	89 (21.9)	
4. “I’m okay unless something goes wrong”	305 (36.9)	164 (39.1)	156 (38.4)	
5. “I’m in control and manage well”	210 (25.4)	112 (26.7)	144 (35.5)	
**How I feel about managing my daily activities**				
Total responses	826	417	405	
Median response	3.5 (3.0, 4.0)	4.0 (3.0, 4.0)	4.0 (3.0, 5.0)	< 0.001**
1. “I don’t feel able to manage”	19 (2.3)	4 (1.0)	3 (0.7)	
2. “It’s a struggle and I get a lot of help”	79 (9.6)	34 (8.2)	24 (5.9)	
3. “I get some help from other people”	315 (38.1)	153 (32.4)	116 (28.6)	
4. “I’m okay unless something goes wrong”	241 (29.2)	135 (32.4)	127 (31.4)	
5. “I’m in control and manage well”	172 (20.8)	91 (21.8)	135 (33.3)	
**Fear of falling**				
Total responses	827	418	405	
Median response	4.0 (3.0, 4.0)	4.0 (3.0, 4.0)	4.0 (3.0, 4.0)	< 0.001**
1.“I hardly go outside now”	81 (9.8)	24 (5.7)	14 (3.5)	
2.“I have changed a lot of my activities”	118 (14.3)	56 (13.4)	25 (6.2)	
3. “I have changed some of my activities”	193 (23.3)	94 (22.5)	66 (16.3)	
4. “I worry but won’t let it stop me”	375 (45.3)	208 (49.8)	235 (58.0)	
5. “I have no fear of falling”	60 (7.3)	36 (8.6)	65 (14.9)	
**My confidence when walking outside**				
Total responses	828	419	405	
Median response	3.0 (2.0, 4.0)	3.0 (3.0, 4.0)	4.0 (3.0, 4.0)	< 0.001**
1. “I hardly go outside now”	56 (6.8)	20 (4.8)	16 (4.0)	
2. “I have to take someone to help me”	176 (21.3)	72 (17.2)	43 (10.6)	
3. “I only go familiar routes”	233 (28.1)	133 (31.7)	92 (22.7)	
4. “I get nervous sometimes”	264 (31.9)	149 (35.6)	160 (39.5)	
5. “I’ve got no problem walking outside”	99 (12.0)	45 (10.7)	94 (23.2)	
**My social network**				
Total responses	828	419	404	
Median response	4.0 (3.0, 5.0)	4.0 (3.0, 5.0)	4.0 (4.0, 5.0)	0.078
1. “I’m alone all the time”	16 (1.9)	2 (0.5)	6 (1.5)	
2. “I’m alone frequently”	97 (11.7)	39 (9.3)	31 (7.7)	
3. “I’m alone sometimes”	163 (19.7)	85 (20.3)	60 (14.9)	
4. “I’ve got a few good friends”	298 (36.0)	152 (36.3)	162 (40.1)	
5. “I’ve got lots of friends and relations”	254 (30.7)	141 (33.7)	145 (35.9)	

*IQR* Interquartile range

^**^P < 0.001

##### Completers versus dropouts

The same regression model, after removal of declined referrals from the non-completers group (i.e., completers versus dropouts), captured 46.1% (*n* = 385) of cases. The model was significant (*P* < 0.001), explained between 9 (Cox & Snell R square) and 12% (Nagelkerke R squared) of variation in status, and accurately classified 65% of cases. The same four variables remained after backward elimination; compared to completers, participants who started Staying Steady before dropping out were more likely to be prescribed oral nutritional support (*n* = 767; RR 10.89, 95% CI 1.39, 85.56), diagnosed with COPD (*n* = 736; RR 2.74, 95% CI 1.61, 4.68), and have a high fear of falling (*n* = 583; RR 1.58, 95% CI 1.23, 2.01). The RR for stroke or transient ischaemic attack was non-significant (*n* = 736; 1.33, 95% CI 0.99, 1.78).

##### Completers versus declined referral

In addition to variables excluded from previous regression models (BMI, self-referral questionnaire responses and goal setting; Sect. 2.5), ethnicity, use of a walking aid at baseline and PROMs were also excluded from this model due to > 40% missing data. The model captured 56.4% of cases (*n* = 483), was significant (*P* < 0.001), explained between 9 (Cox & Snell R square) and 13% (Nagelkerke R squared) of variation in status, and accurately classified 77% of cases. After backward elimination five variables remained: registered disability (*P* = 0.045), self-identified disability (*P* < 0.001), prescribed oral nutritional support (*P* = 0.999), being prescribed statins (*P* = 0.080) and IMD rank (*P* < 0.001). People who declined a referral were more likely than completers to be prescribed oral nutritional support (*n* = 788; RR 11.41, 95% CI 1.47, 88.73) and live in an area of high deprivation (*n* = 824; RR 1.43, 95% CI 1.23, 1.66) and less likely to consider themselves disabled (*n* = 601; RR 0.68, 95% CI 0.53, 0.88). The risk for statin prescription (*n* = 789; RR 0.97, 95% CI 0.86, 1.09) or registered disability (*n* = 604; RR 1.04, 95% CI 0.77, 1.40) were non-significant.

#### Patient reported outcome measures

Participants reported an improvement in their ability to manage their health and daily activities, fear of falling and confidence when walking outside, but not in their social network (Table 4).

#### Goal setting and evaluation

Goals set at the start of Staying Steady are shown in Table 2. Upon evaluation, completers of Staying Steady achieved their goals through the programme completely (*n* = 224; 60.4%), partially (*n* = 128; 34.5%) or not at all (*n* = 19; 5.1%). Most participants reported that Staying Steady made a difference to them (*n* = 392; 95.8%); fourteen (3.4%) felt that completing Staying Steady made no difference and three (0.7%) were unsure. The education sessions were considered useful by 352 (92.6%) participants, compared to 17 (4.5%) and 11 (2.9%) who did not find the education useful, or did not receive education, respectively. The difficulty of the exercises was considered just right (*n* = 273; 94.8%), too easy (*n* = 4; 1.4%) or too hard (*n* = 11; 3.8%), and most responses indicated the exercises were progressive (*n* = 334; 87.4%). Most completers planned to continue exercising (*n* = 361; 94.0%). Thirteen participants (3.4%) were unsure, and ten (2.6%) had no plans to continue exercising. Most completers would recommend Staying Steady to others (*n* = 405; 98.5%).

## Discussion

This service evaluation aimed to assess the effectiveness of the Healthworks Staying Steady falls prevention programme to improve physical function and PROMs, using routinely collected data. We also assessed goal setting and evaluation of the programme by completers. Primary findings show significant improvements in the 30-s CS, TUG and 4SBT performance. Improvements in these outcome measures are beneficial, as poor physical function is associated with greater dependence in activities of daily living in older adults [38]. Most improvements in physical function remained significant after results were stratified by presence of chronic diseases except for HF and COPD, where fewer improvements in physical function were observed. Factors impacting attrition included presence of COPD, prescribed oral nutritional support, fear of falling, social deprivation and self-identified disability.

### Primary outcomes

#### 30-s chair stand test

Greater leg strength is associated with improved quality of life [39] and reduced fall risk [40]. We reported improved median CS performance in participants who completed the Staying Steady programme. In comparison, others report no difference in CS performance between patients who attended a 16-week falls prevention programme embedded in primary care, compared to a usual care control group [41]. Notably, the multicomponent falls prevention programme assessed by Siegrist and colleagues dedicated six, one-hour sessions to strengthening exercises over the 16-week intervention period [41]. A strength training component was included in every Staying Steady exercise session, this may indicate the importance of adequate training volume to gain significant improvements in physical function in adults at risk of falling.

Although statistically significant, we report median improvements in CS not exceeding the MDC of 3.9 repetitions [32]. The proportion of participants demonstrating an improvement greater than the MDC ranged between 24 and 41% for the CS. However, the magnitude of the effect is likely to be underestimated in the 94 participants who needed a walking aid at baseline, but not follow-up. Enabling someone to stand unaided, when they were previously unable to, is likely to have a meaningful impact on their quality of life which is not captured by the number of repetitions completed in a specified time. In this context, the change in CS ability might still be considered meaningful for these participants, despite the increase in repetitions falling short of the MDC.

Twenty-two participants completed the CS unaided at baseline but with assistance at follow-up. The introduction of walking aids after participants were previously able to stand independently suggests declining functional performance, which could translate to a loss of independence in everyday life. Possible reasons for this can be speculated to be deteriorating health, loss of confidence or inconsistent judgement, or instructions from supervising practitioners.

#### Timed up and go test

Poor performance in the TUG predicts adverse health outcomes in older adults [26]. Meaningful improvements in the TUG were achieved by participants who completed both assessments unaided and who needed a walking aid at baseline only. Improvements greater than the MDC were achieved by 70.0 and 93.8%, respectively [33]. No statistical improvement in TUG time was seen in thirteen participants who used a walking aid at both timepoints, although the small sample size limits the certainty of this outcome. Interestingly, around half (53.8%) of participants in this group demonstrated an improvement in the TUG greater than the MDC. In comparison, following a previous 16-week falls prevention programme, only 24.6% of participants demonstrated an improvement greater than the MDC despite an improvement of any magnitude being reported in 89.3% of participants [11]. The greater proportion of participants in the present evaluation achieving a meaningful improvement might be due to use of different population-specific estimates for the MDC. Alternatively, the longer duration of the Staying Steady programme might facilitate greater improvements in physical function.

#### Four-stage balance test

In the present evaluation, median balance score increased from level two to level three, indicating a reduction in number of participants at high risk of falling [28]. Others report that higher baseline Activities-specific Balance Confidence (ABC) Scale score predicted improvements in 4SBT level following a falls prevention intervention [42]. The 16-item ABC Scale captures the participants self-reported confidence in maintaining their balance and stability in various everyday environments, including walking outside the house and transferring to or from a car [43]. Although the ABC Scale was not used in this evaluation, completers of Staying Steady reported improved confidence when walking outside and reduced fear of falling (Table 4), which might infer improved balance confidence. Although only baseline confidence was previously associated with improved balance [42], presently both confidence-related PROMs and 4SBT score were improved post-intervention. Therefore, it is unclear whether better balance is consequential of improved confidence or vice versa in this cohort.

### Impact of chronic disease on functional outcomes

Improved CS ability was evident for most subgroups when stratified by chronic diseases, excluding in people with HF and COPD. Sub-group analyses for the unaided TUG remained significant for all groups. Finally, 4SBT scores remained significant when results were stratified by chronic diseases, except in participants with HF. Due to the small sample of participants with HF and COPD, it is difficult to make inferences into the reason for this lack of change.

### Factors associated with non-completion

Regular exercise attenuates age-related deterioration of muscle strength [44], and reduces falls [45], morbidity, and mortality risk [46]. To successfully recruit and retain older adults into exercise interventions, we need to understand the factors that influence participation.

People who declined a referral more frequently lived in an area of high deprivation than completers of Staying Steady [35]. The influence of social deprivation on poor exercise uptake is likely to be multifactorial [47, 48]. Importantly, low socioeconomic status is associated with increased mortality risk [49] and an exaggerated loss in age-related physical function [50] compared to higher socioeconomic status, indicating a greater need for intervention in the former group. Therefore, the results of this study indicate a perpetuation of the Inverse Care Law, whereby health-related interventions are accessed least by those with the greatest need [51]. However, the referred participant’s perception of their need for intervention is also important to consider. We found that people who declined a referral were less likely than completers to consider themselves disabled. Mobility-related physical disability in older adults can be preventable [52]. Thus, falls prevention interventions are often implemented to minimise the impact of disability or dependency in everyday activities. Therefore, the decision to decline a referral might result from a perception of the intervention as unnecessary if the participant is already able to live independently.

Presence of COPD was associated with dropping out of Staying Steady. Low quadricep strength [53] and aerobic capacity [54] increase mortality risk in people with COPD. Both variables can potentially be improved with exercise [55], highlighting the importance of encouraging exercise uptake in these patients. In addition, fear of falling and prescribed oral nutritional support influenced non-completion of Staying Steady. Both undernutrition and fear of falling are associated with the frailty phenotype [56], suggesting that the most frail participants are more likely to drop out of exercise interventions. Importantly, frailty can be prevented with regular exercise participation [56]. Therefore, strategies to retain these participants in long-term exercise programmes should be investigated.

### Acceptability of the intervention

Uptake on to Staying Steady (~ 67% of appropriately referred participants) was lower than average uptake (81%) of exercise referral schemes in Northumberland, UK [57]. The reason for most declined referrals was not recorded (41%). The most cited reason for declining a referral was that the participant did not want to engage with the service (Fig. 1). Potential reasons for this lack of engagement have been discussed. Evaluation of Staying Steady by completers was overwhelmingly positive and no adverse events associated with Staying Steady were recorded during the evaluation period. However, the reason for most participant drop-outs is unknown (65%). In the absence of follow-up data for non-completers, we cannot exclude that some participants might have dropped out following an adverse intervention effect. The observed dropout, by our definition of completing measurements at baseline but not at follow-up, was similar to Orton and colleagues [12] who reported 348 people at baseline and 203 at follow-up.

### Strengths and limitations

This evaluation involves a large sample of data collected during routine practice, representing the effectiveness of Staying Steady at a local level. Recent service evaluations have demonstrated the effectiveness of falls prevention programmes based on the FaME intervention to improve physical function [11, 12]. The present study complements and extends the findings of existing service evaluations, by providing novel insight into the influence of morbidity on outcome measures and into demographic characteristics influencing attrition and adherence.

Limitations include the risk of selection bias that could result in an over-estimation of the effect of the Staying Steady programme. The nature of a retrospective service evaluation is that follow-up data on those who dropped out is unavailable, therefore the effect of the intervention in this group is unknown. However, the intervention was effective in those that completed the programme and we have been able to identify characteristics of those more likely to drop out. The latter can be used to identify the people more likely to drop out to better understand how the service can be changed to meet their needs. Another potential source of bias could be missing data, however, this issue is mitigated by absent data being missing completely at random [30], and therefore unlikely to introduce bias. We acknowledge that some outcome measures may not be tested as rigorously as we would expect in a controlled study, for example, the use of hands to assist with the chair stand may not be accepted methods observed in controlled trials. However, we consider our findings to be pragmatic and more realistic of the target population, as recruitment of older adults with functional limitations to community-delivered exercise programmes is of utmost importance. Furthermore, our findings reflect standard practice in community-delivered exercise programmes allowing us to highlight good practices and recognise areas that require further consideration. Finally, Healthworks aimed to deliver 50 h of exercise intervention over 27 weeks. Approximately 25 h were expected to be undertaken at home. This was not monitored so compliance cannot be determined. This may explain some of the variation in responses to the exercise programme.

### Implications for practice and future research

Overall, Staying Steady appears an effective community-based initiative to engage older adults in falls prevention exercises, resulting in positive outcomes and no reported safety issues. Future research should investigate strategies to encourage adherence in people from areas of high deprivation, with COPD and presenting with frailty-related issues. In practice, continued compliance with guidelines for falls prevention programmes is recommended. Guidelines recommend flexibility in programme delivery to accommodate participant needs [3]. The importance of adherence to this guideline in practice is demonstrated by the lack of improvement in outcome measures seen sub-groups of participants with HF and COPD.

## Conclusion

The Healthworks Staying Steady exercise programme improved 30-s CS, TUG and 4SBT performance, in a mixed morbidity cohort of older adults at risk of falling in the Northeast of England. High satisfaction with the programme is evident through participant evaluation of Staying Steady. However, the reasons for a lack of improvement in primary outcomes for people with HF and COPD should be further investigated. Finally, efforts to recruit and retain participants from groups associated with low uptake and adherence are essential.

## Supplementary Information

**Additional file 1: Supplementary Material 1.** Goal setting questionnaires provided to participants at the start of the Healthworks Staying Steady 27-week falls prevention programme. **eTable 1.** Patient reported outcome measures completed by participants of the Healthworks Staying Steady 27-week falls prevention programme. **eTable 2.** Follow-up questionnaires given to participants who completed the Healthworks Staying Steady 27-week falls prevention exercise programme. Follow-up questionnaires and answers were updated from 2017 and are therefore, presented separately by date. **eTable 3.** Baseline and follow-up physical function data for participants who completed the Staying Steady 27-week falls prevention programme, stratified by presence of chronic disease.

## Data Availability

The data sets analysed during the current study are available from the corresponding author on reasonable request and with permission of Healthworks. The data sets analysed during the current study are not publicly available because ethics approval for this service evaluation was granted with the condition that published data would be generalised and individual participant data would not be published.

## Abbreviations

4SBT: Four-stage balance test
ABC: Activities-specific Balance Confidence
BMI: Body mass index
CHD: Coronary heart disease
CI: Confidence interval
CS: Chair stand
FaME: Falls Management Exercise
FRAT: Falls Risk Assessment Tool
IMD: Index of Multiple Deprivation
IQR: Interquartile range
MDC: Minimal detectable change
NHS: National Health Service
NUTH: Newcastle upon Tyne Hospitals
PROMs: Patient reported outcome measures
RR: Relative risk
TUG: Timed up and go
UK: United Kingdom
USA: United States of America
