# Supplementary material for: The effectiveness of the Healthworks Staying Steady community-based falls prevention exercise programme to improve physical function in older adults: a 6-year service evaluation
Source: BMC Public Health. 2022 Aug 1;22:1457. doi: 10.1186/s12889-022-13832-3 (PMC9341056; doi:10.1186/s12889-022-13832-3)
Supplement: Supplementary file 1 — Additional file 1: Supplementary Material 1. Goal setting questionnaires provided to participants at the start of the Healthworks Staying Steady 27-week falls prevention programme. eTable 1. Patient reported outcome measures completed by participants of the Healthworks Staying Steady 27-week falls prevention programme. eTable 2. Follow-up questionnaires given to participants who completed the Healthworks Staying Steady 27-week falls prevention exercise programme. Follow-up questionnaires and answers were updated from 2017 and are therefore, presented separately by date. eTable 3. Baseline and follow-up physical function data for participants who completed the Staying Steady 27-week falls prevention programme, stratified by presence of chronic disease. [file 12889_2022_13832_MOESM1_ESM.docx]

The effectiveness of the Healthworks Staying Steady community-based falls prevention exercise programme to improve physical function in older adults: A 6-year service evaluation.

Emily James^1^, Paul Oman^2^, Michael Ali^3^, Paul Court^3^, Stuart Goodall^1^, Simon J Nichols^4,5^, Alasdair F O’Doherty^1^

1. Department of Sport, Exercise and Rehabilitation, Northumbria University, Newcastle-Upon-Tyne, UK

2. Department of Mathematics, Physics and Electrical Engineering, Northumbria University, Newcastle-Upon-Tyne, UK

3. Healthworks, Newcastle-Upon-Tyne, UK

4. Sport and Physical Activity Research Group, Sheffield Hallam University, Sheffield, UK

5. Advanced Wellbeing Research Centre, Sheffield Hallam University, Sheffield, UK

**Additional File 1**

Contents:

Supplementary Material 1: Goal setting questionnaires provided to participants at the start of the Healthworks Staying Steady 27-week falls prevention programme.

eTable 1. Patient reported outcome measures completed by participants of the Healthworks Staying Steady 27-week falls prevention programme.

eTable 2. Follow-up questionnaires given to participants who completed the Healthworks Staying Steady 27-week falls prevention exercise programme. Follow-up questionnaires and answers were updated from 2017 and are therefore, presented separately by date.

eTable 3. Baseline and follow-up physical function data for participants who completed the Staying Steady 27-week falls prevention programme, stratified by presence of chronic disease.

**Supplementary Material 1: Goal setting questionnaires provided to participants at the start of the Healthworks Staying Steady 27-week falls prevention programme.**

Two versions of the questionnaire are provided, relating to the format used prior to 2017 and from 2017 onwards.

**Goal setting questionnaire used by Healthworks for the Staying Steady programme prior to 2017**

Participants are asked to choose one goal from the following:

1. I would like to reduce my fear of falling
2. I would like to feel more stable
3. I would like to feel fitter
4. I would like to feel stronger
5. I would like to feel more confident when taking public transport ^a^
6. I would like to feel more confident when walking outside ^a^
7. I would like to socialise more
8. I would like to feel more able to manage my health

**Goal setting questionnaire used by Healthworks for the Staying Steady programme from 2017 onwards**

Participants can choose as many as they wish from the following:

1. I would like to reduce my fear of falling
2. I would like to feel more stable
3. I would like to feel stronger
4. I would like to feel more confident out and about
5. I would like to socialise more

Note. Where previously assessed items were later removed from standard practice, these variables were excluded from analysis or grouped with the most similar equivalent in the updated format.

^a^ Goals denoted here were grouped under “I would like to feel more confident out and about”, to reflect goal number four in the most recent delivery format of Staying Steady.

**eTable 1. Patient reported outcome measures (“Current situation”) completed by participants of the Healthworks Staying Steady 27-week falls prevention programme.**

| **Domain** | **Responses prior to 2017** | **Responses from 2017 onwards** |
| --- | --- | --- |
| Managing my health | 1. I don’t feel able to manage 2. It’s a struggle but I get by with help 3. I manage with help from others 4. Unless something goes wrong, I manage well 5. I feel in control and I manage well | 1. I don’t feel able to manage 2. It’s a struggle and I get a lot of help 3. I get some help from other people 4. I’m okay unless something goes wrong 5. I’m in control and manage well |
| Activities of daily living | 1. Very poor 2. Poor 3. Average 4. Good 5. Excellent | 1. I don’t feel able to manage 2. It’s a struggle and I get a lot of help 3. I get some help from other people 4. I’m okay unless something goes wrong 5. I’m in control and manage well |
| Fear of falling | 1. Very frightened and I hardly go outside 2. I worry a lot and always think about it 3. I’ve changed some of my activities, but I am ok 4. I worry about it but it doesn’t stop my life 5. I’m not afraid of falling | 1. I hardly go outside now 2. I have changed a lot of my activities 3. I have changed some of my activities 4. I worry but won’t let it stop me 5. I have no fear of falling |
| My confidence when walking outside | 1. Very poor 2. Poor 3. Average 4. Good 5. Excellent | 1. I hardly go outside now 2. I have to take someone to help me 3. I only go on familiar routes 4. I get nervous sometimes 5. I’ve got no problems walking outside |
| My social network | 1. I feel alone all the time 2. I feel alone frequently 3. I feel alone sometimes 4. I feel connected to a few key people 5. I feel connected to lots of people | 1. I’m alone all the time 2. I’m frequently alone 3. I’m sometimes alone 4. I’ve got a few good friends 5. I’ve got lots of friends and relations |

Note. Participants selected one Likert-scale response for each domain from the centre column (prior to 2017) or right column (2017 onwards). Pre-and post- intervention PROMs are presented in Table 4 in the main text. For analysis, responses given prior to 2017 are grouped with their corresponding number in the most recent delivery format.

**eTable 2. Follow-up questionnaires given to participants who completed the Healthworks Staying Steady 27-week falls prevention exercise programme.**

| Question | Answers |
| --- | --- |
| **Prior to 2017** |  |
| Overall, do you feel you have benefited from the Staying Steady programme? ^a^ | - Yes - No |
| Did you achieve the goals that you set at the start of the programme? ^b^ | - Yes - No |
| Do you have plans to continue exercising? | - Yes - No |
| Would you recommend the Staying Steady exercise classes to your friends? ^c^ | - Yes - No |
| Do you feel the exercises got progressively harder throughout the programme? ^d^ | - Yes - No |
| **From 2017 onwards** |  |
| Have you achieved what you wanted to at the start of the programme? ^b^ | - Yes - No - Partially |
| Do you feel Staying Steady has made a difference to you? ^a^ | - Yes - No |
| How do you feel it has made a difference | - Free text box |
| Did you find the education sessions useful? | - Yes - No |
| Were the exercises: | - Too hard - Too easy - Just right |
| Did they get progressively harder? ^d^ | - Yes - No |
| Would you recommend Staying Steady to someone else? ^c^ | - Yes - No |

Note. Follow-up questionnaires and answers were updated from 2017 and are therefore, presented separately by date. Where previously assessed items were later removed from standard practice, these variables were excluded from analysis or grouped with the most similar equivalent in the updated format. ^a, b, c, d^ For analysis, variables used prior to 2017 and denoted with a letter were grouped with the corresponding letter variable from 2017 onwards.

**eTable 3 Baseline and follow-up physical function data for participants who completed the Staying Steady 27-week falls prevention programme, stratified by presence of chronic disease.**

| **Outcome** |  | **Chronic disease** | | | | | | | |
| --- | --- | --- | --- | --- | --- | --- | --- | --- | --- |
|  | All | Cardiac disease | Coronary heart disease | Heart failure | Risk for coronary heart disease | Stroke / transient ischaemic attack | Chronic obstructive pulmonary disease | Osteoporosis / osteopenia | Chronic kidney disease |
| **Chair stand (reps) *Unaided*** |  |  |  |  |  |  |  |  |  |
| n | 264 | 69 | 39 | 6 | 105 | 43 | 7 | 41 | 71 |
| Baseline | 8.0 (6.0, 10.0) | 8.0 (6.0, 10.0) | 8.0 (6.0, 10.0) | 8.5 (4.8, 10.3) | 8.0 (6.0, 10.0) | 8.0 (6.0, 10.0) | 9.0 (7.0, 11.0) | 7.0 (5.0, 9.0) | 8.0 (6.0, 10.0) |
| Follow-up | 11.0 (9.0, 13.0) | 11.0 (9.0, 13.0) | 11.0 (8.0, 12.0) | 10.5 (9.5, 12.3) | 11.0 (9.0, 12.5) | 10.0 (9.0, 12.0) | 10.0 (9.0, 11.0) | 11.0 (9.0, 12.0) | 11.0 (10.0, 12.0) |
| P | <0.001** | <0.001** | <0.001** | 0.063 | <0.001** | <0.001** | 0.125 | <0.001** | <0.001** |
| **Chair stand (reps)**  ***Aided*** |  |  |  |  |  |  |  |  |  |
| n | 54 | 19 | 15 | 3 | 18 | 11 | 3 | 10 | 15 |
| Baseline | 7.0 (5.0, 9.0) | 7.0 (6.0, 9.0) | 7.0 (6.0, 10.0) | 5.0 (4.0, 7.0) | 6.0 (5.0, 8.3) | 7.0 (6.0, 10.0) | 12.0 (5.0, 12.0) | 7.0 (5.8, 9.3) | 7.0 (5.0, 9.0) |
| Follow-up | 9.0 (7.0, 11.0) | 10.0 (7.0, 12.0) | 10.0 (9.0, 13.0) | 5.0 (4.0, 8.0) | 9.0 (7.0, 11.3) | 9.0 (8.0, 12.0) | 12.0 (4.0, 16.0) | 10.0 (8.0, 10.3) | 10.0 (8.0, 13.0) |
| P | <0.001** | 0.004** | 0.022* | 1.00 | <0.001** | 0.016* | 1.00 | 0.070 | <0.001** |
|  |  |  |  |  |  |  |  |  |  |
| **Chair stand (reps)**  ***Aided at baseline only*** |  |  |  |  |  |  |  |  |  |
| n | 94 | 25 | 17 | 2 | 41 | 10 | 6 | 16 | 25 |
| Baseline | 8.5 (7.0, 10.0) | 8.0 (6.5, 11.0) | 8.0 (6.0, 11.0) | 5.5 (3.8, 6.3) | 8.0 (6.0, 10.0) | 7.5 (5.8, 9.3) | 10.0 (6.5, 12.3) | 8.5 (6.3, 10.8) | 8.0 (7.0, 9.5) |
| Follow-up | 10.0 (9.0, 13.0) | 10.0 (9.0, 13.0) | 10.0 (9.0, 12.5) | 10.0 (7.5, 9.5) | 10.0 (8.0, 12.5) | 8.0 (7.0, 9.3) | 12.5 (10.0, 18.3) | 11.0 (8.3, 13.0) | 10.0 (8.5, 13.0) |
| P | <0.001** | 0.001** | 0.002** | 0.500 | <0.001** | 0.344 | 0.031* | 0.001** | <0.001** |
| **Chair stand (reps) *Aided at follow-up only*** |  |  |  |  |  |  |  |  |  |
| n | 22 | 12 | 5 | 3 | 6 | 3 | 2 | 3 | 6 |
| Baseline | 7.5 (5.0, 9.0) | 8.0 (5.5, 9.0) | 7.0 (4.0, 8.0) | 8.0 (4.0, 8.0) | 5.5 (4.8, 6.8) | 9.0 (5.0, 10.0) | 6.5 (3.8, 8.3) | 8.0 (4.0, 9.0) | 6.5 (3.8, 8.3) |
| Follow-up | 10.0 (7.8, 12.0) | 11.0 (7.5, 12.8) | 11.0 (9.0, 12.5) | 5.0 (5.0, 11.0) | 9.5 (7.3, 10.3) | 10.0 (8.0, 12.0) | 9.0 (5.3, 11.3) | 7.0 (5.0, 14.0) | 9.0 (5.0, 11.0) |
| P | <0.001** | 0.012* | 0.063 | 1.00 | 0.031* | 0.250 | 0.500 | 1.00 | 0.219 |
| **TUG (s)**  ***Unaided*** |  |  |  |  |  |  |  |  |  |
| n | 387 | 113 | 69 | 12 | 149 | 52 | 16 | 61 | 104 |
| Baseline | 13.0 (10.5, 16.4) | 13.2 (11.0, 16.7) | 13.3 (11.7, 18.0) | 15.1 (11.0, 17.2) | 12.9 (10.2, 16.6) | 15.0 (11.9, 18.5) | 11.6 (9.6, 16.5) | 14.0 (11.0, 18.0) | 13.4 (10.6, 17.0) |
| Follow-up | 9.7 (8.0, 11.9) | 10.0 (8.1, 11.9) | 10.7 (8.1, 12.3) | 10.8 (9.5, 12.0) | 9.5 (8.0, 11.6) | 10.9 (8.6, 14.0) | 8.6 (6.5, 9.5) | 9.9 (8.2, 12.1) | 10.2 (8.2, 11.9) |
| P | <0.001** | <0.001** | <0.001** | 0.006** | <0.001** | <0.001** | <0.001** | <0.001** | <0.001** |
| **TUG (s)**  ***Aided*** |  |  |  |  |  |  |  |  |  |
| n | 13 | 3 | 1 | 1 | 7 | 2 | 0 | 2 | 5 |
| Baseline | 22.7 (15.3, 26.7) | 22.7 (17.5,49.2) | / | / | 25.0 (13.8, 28.0) | 42.9 (10.3, 56.0) | / | 38.6 (21.0, 38.8) | 25.3 (18.2, 60.6) |
| Follow-up | 16.0 (13.0, 23.0) | 27.0 (12.0, 55.8) | / | / | 16.0 (16.0, 18.9) | 17.4 (12.0, 15.7) | / | 37.3 (14.2, 43.4) | 27.0 (17.4, 43.1) |
| P | 0.221 | 1.00 | / | / | 0.453 | 1.00 | / | 1.00 | 1.00 |
| **TUG (s)**  ***Aided at baseline only*** |  |  |  |  |  |  |  |  |  |
| n | 32 | 7 | 4 | 0 | 14 | 10 | 2 | 7 | 5 |
| Baseline | 18.0 (15.0, 23.6) | 15.7 (14.6, 17.1) | 16.8 (9.9, 17.4) | / | 19.9 (15.5, 30.8) | 15.6 (14.0, 19.3) | 18.4 (11.0, 19.0) | 18.0 (16.3, 18.7) | 17.5 (12.5, 19.5) |
| Follow-up | 12.3 (10.0, 14.6) | 12.4 (10.8, 14.0) | 11.9 (7.2, 13.6) | / | 12.1 (9.8, 15.5) | 11.1 (9.7, 13.5) | 14.4 (7.5, 15.6) | 12.4 (11.5, 13.6) | 13.6 (9.1, 14.2) |
| P | <0.001** | 0.016* | 0.125 | / | <0.001** | 0.002** | 0.500 | 0.016* | 0.063 |
| **4SBT (level)** |  |  |  |  |  |  |  |  |  |
| n | 295 | 71 | 37 | 9 | 116 | 46 | 16 | 46 | 75 |
| Baseline | 2.0 (2.0, 3.0) | 2.0 (2.0, 3.0) | 2.0 (2.0, 3.0) | 2.0 (1.5, 3.0) | 2.0 (2.0, 3.0) | 2.0 (2.0, 3.0) | 3.0 (2.0, 3.0) | 2.0 (2.0, 3.0) | 2.0 (2.0, 3.0) |
| Follow-up | 3.0 (3.0, 4.0) | 3.0 (3.0, 4.0) | 3.0 (3.0, 3.0) | 3.0 (2.0, 3.0) | 3.0 (3.0, 4.0) | 3.0 (3.0, 3.0) | 3.0 (3.0, 4.0) | 3.0 (3.0, 4.0) | 3.0 (3.0, 4.0) |
| P | <0.001** | <0.001** | <0.001** | 0.727 | <0.001** | <0.001** | 0.006** | <0.001** | <0.001** |

Note. Participants were evaluated separately based on whether they used an aid to perform the test (including a walking aid or used their hands to push themselves up). Values are median (interquartile range). 4SBT= Four-stage balance test; TUG = Timed Up and Go. Participants were grouped based on whether they used arms to chair-stand or used a walking-aid at both timepoints (‘aided’), neither timepoint (‘unaided’), at baseline but not follow-up (‘aided at baseline only’), or at follow-up but not baseline (‘aided at follow-up only’). / Denotes insufficient cases for analyses. *P <0.05. between baseline and follow-up. **P <0.01 between baseline and follow-up.
